# Legal and Ethical Challenges in Developing a Dutch Nationwide Hepatitis C Retrieval Project (CELINE)

**DOI:** 10.34172/ijhpm.2020.33

**Published:** 2020-03-07

**Authors:** Marleen van Dijk, Frans M. van Agt, Joost P.H. Drenth

**Affiliations:** ^1^Department of Gastroenterology & Hepatology, Radboud University Medical Centre, Nijmegen, The Netherlands.; ^2^Ethics Committee ‘CMO ArnhemNijmegen,’ Nijmegen, The Netherlands.

**Keywords:** Hepatitis C Elimination, Retrieval, Legal Barriers, Ethical Barriers, Linkage to Care

## Abstract

In 2016 the World Health Organization (WHO) called upon nations worldwide to eliminate viral hepatitis. Due to suboptimal hepatitis C virus (HCV) therapies in the past, many patients could not be treated or cured. With the current options, all patients can be treated and >90% is cured. However, these developments have not reached all patients, especially those who were lost to follow-up (LTFU) in previous years, an estimated 30% in the Netherlands. Retrieving these patients can contribute to HCV elimination. In light of this, we aimed to develop a nationwide retrieval strategy. During development we identified four major challenges. The first challenge is ethical and arises from the aim of the project: should physicians retrieve LTFU patients? We argue that the arguments in favour outweigh those against. The three other challenges are methodological and mainly legal in nature. Firstly, how far back are we allowed to trace LTFU patients? In the Netherlands, patient files should be kept for a minimum of fifteen years, but in chronic disease they may be archived longer. Secondly, which professional should identify the LTFU patients? Ideally this would be the treating physician, but we describe the circumstances that allow inclusion of assistance. Lastly, what is the proper way to invite the LTFU patients? We found that we can often request current address information from municipalities, and explain this process in detail. The offered solutions are feasible and translatable to other healthcare environments. We hope to take away any insecurities people may have about the ethical and legal nature of such a retrieval project and hope to inspire others to follow in our footsteps.

## Introduction

Hepatitis C virus (HCV) infection is a cause of liver disease that becomes chronic in 70%-75% of cases. Infection may result in life-threatening complications such as cirrhosis, hepatocellular carcinoma, and death. With 71 million people affected worldwide, global annual HCV mortality has increased in the past 15 years.^1^ In 2016, the World Health Organization (WHO) has set viral hepatitis elimination goals, which call for a 90% reduction in new chronic infections and a 65% reduction in mortality by 2030. Numerous countries, including the Netherlands, have agreed to comply with these goals. A Dutch national hepatitis plan was developed in 2016, focusing on five key areas of interest: (1) awareness and vaccination, (2) identification of infected patients, (3) diagnostics and treatment, (4) improved organization of hepatitis care and (5) surveillance of identified patients.

The third key area is of particular interest to us: diagnostics and treatment. Until 2014, the standard of care for chronic HCV patients was pegylated interferon with ribavirin, a lengthy, moderately effective and ill-tolerated treatment which cured only 40%-80% of patients. As a result, many patients had no treatment options or declined receiving treatment. In the Netherlands, it is estimated that ~30% of all diagnosed HCV patients have disappeared from care (lost to follow-up: LTFU).^2,3^ The advent of direct acting antivirals (DAAs) in 2014 completely changed the therapeutic landscape of HCV. With an average treatment duration of 8-12 weeks, cure rates are >90%. The only remaining challenging group are patients with decompensated cirrhosis.^4^ Unfortunately, these novel therapeutic developments have not reached many LTFU patients. They are still at risk for liver related complications and would benefit greatly from reassessment. This calls for a systematic search for the LTFU population: retrieval.^5^

In the past decade, numerous regional HCV retrieval projects have been carried out in the Netherlands. CELINE (‘Hepatitis C elimination in the Netherlands’) is the first nationwide approach and was developed based on these regional projects. This paper outlines the CELINE retrieval strategy which comes with various ethical and legal challenges. We aim to offer solutions and provide a legal framework for clinicians and researchers interested in retrieval.

## CELINE Methodology

As described in Figure, CELINE consists of four phases. In the first phase, laboratory records and patient charts are reviewed to identify patients who are LTFU. HCV antibody tests, Western blots, RNA tests, and genotyping results are reviewed. We identify patients who are possibly chronically infected and patients who were chronically infected at the time of the last test. The first group consists of patients who have a positive anti-HCV test without a known RNA result. The second group consists of patients in whom the last RNA result was positive. Patients records from both groups will be reviewed to ascertain whether they are LTFU.

**Figure:**
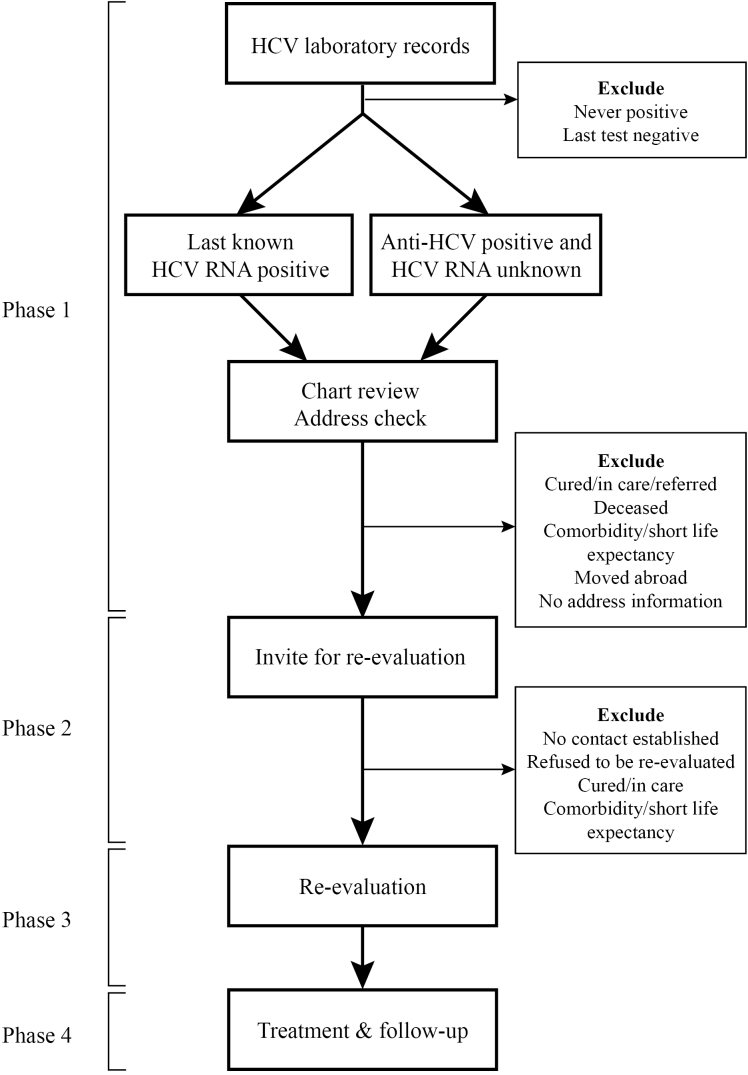
Methodology of CELINE. Abbreviations: HCV, hepatitis C virus; CELINE, Hepatitis C elimination in the Netherlands.

The review of laboratory and patient records results in a cohort of LTFU patients. Patients residing in the Netherlands are invited via letter to be re-evaluated during phase 2. Patients who are 18 years or older are furthermore informed about the CELINE research project, which consists of patient file research. Patients are contacted by phone 1-2 weeks afterwards to ascertain if they want to be re-evaluated. When patients do wish to be re-linked to care, their general practitioner is contacted and asked for a referral letter.

Phase 3 consists of re-evaluating the LTFU patients. This re-evaluation is part of standard clinical care and serves no research purpose. If the patient wants to participate in the CELINE research project, consisting of patient file research, informed consent will be signed during this visit. Data on patient and disease characteristics and retrieval results of patients who have signed informed consent will be collected during phase 4. This data is pseudonymized and stored in a validated and Good Clinical Practice compliant web-based data management program, Castor EDC. Only the local physicians and/or researchers will have access to the local source file linking the codes to specific patients.

The primary outcome of the CELINE research project will be the total number of LTFU patients who have been successfully linked to care. Secondary outcomes include HCV prevalence, number of already successfully treated HCV patients, number of LTFU HCV-positive patients, reasons for LTFU and genotype prevalence, transmission route and liver fibrosis stage (progression) of the LTFU population.

We hypothesize that approximately 25% of ever-diagnosed patients is LTFU and that we will be able to link 25% of invited patients to care again. Estimating a diagnosed population of 16 000, this corresponds to 4000 LTFU patients of whom 1000 will be re-linked to care.

## Legal and Ethical Issues Arising During Development of CELINE

CELINE aims to identify LTFU chronic HCV patients and link them to care. The fundamental question that arises may be defined as follows:

### 1. Should physicians retrieve LTFU patients?

There are several arguments that favour retrieval. Hepatitis C infection causes liver related morbidity and mortality, and as a result an estimated 300 people die each year in the Netherlands.^6^ DAA treatment is fully reimbursed by Dutch healthcare insurance and offering treatment to LTFU patients has many advantages. First, the burden of disease can be lifted from symptomatic patients. Second, treatment curtails development of complications. If patients were retained in care, worsening of their disease would have led to therapy. Since these patients became LTFU, we expect their disease to have progressed further compared to patients who were retained in care. Furthermore, we expect LTFU patients to have more additional risk factors for severe liver disease compared to the population retained in care, such as alcohol or drug use. Treating these patients prevents development of (further) morbidity and mortality, as was shown in a study of Willemse et al.^7^ Treating patients in an early stage of the disease may also offer an economical advantage. Successful treatment will prevent development of both extra-hepatic and hepatic complications, which otherwise would require long-term and costly monitoring and/or treatment.^8^ A systematic review looking at modelling studies performed in the DAA era concluded that scale-up of treatment was generally cost-effective compared to more restrictive treatment.^9^ The fourth advantage of treating LTFU patients is population-based. HCV transmission, though largely limited to specific risk groups in the Netherlands, still occurs in the current day and age. Treatment as prevention is an effective strategy. For example, HIV transmission can be prevented by antiretroviral therapy.^10^ In view of the presence of highly-effective DAAs, *treatment as prevention* is a realistic prospect. This is mostly the case in risk groups with ongoing transmission, such as men who have sex with men (especially HIV-positive men and pre-exposure prophylaxis users) and injecting/intranasal drug users. In this case, retrieval serves to protect uninfected individuals. Finally there is a moral argument: the new therapeutic options are universally effective which is a paradigm with the situation when they left care. Many physicians will feel morally and ethically obliged to inform patients about the greatly improved outlook. Retrieval itself does not violate the patient’s ‘right not to know,’ since they have already received their HCV diagnosis in the past.

However, there are also counter arguments against retrieval. Firstly, since patients are no longer in care, physicians are not contractually obliged to re-establish contact with patients who are not currently being seen in their practice. On the other hand, if the arguments in favour of retrieval outweigh the potential disadvantages one could argue that there is no reason to refrain from participation. Thus, healthcare providers would be technically bound to retrieval, which would result in a situation where non-participating hospitals would be liable. If we did choose to start to retrieve LTFU patients suspected of chronic hepatitis C, we might want to extend retrieval projects to other disorders. However, it is difficult to identify robust criteria for disorders that merit retrieval and we run the risk that the line which distinguishes retrievable from non-retrievable disorders will get blurred easily. Chronic hepatitis C infection only causes complications after a protracted period of time in a limited subset of people. Thus, there is no clinical emergency situation requiring physicians to act immediately in order to avoid an acute and major health hazard. Lastly, retrieval could be seen as a violation of the patient’s autonomy, or a certain ‘right to be left alone.’ This autonomy could be overlooked relative to the public health advantage that retrieval provides. However, it is possible to curtail HCV transmission in other ways, such as education and improving awareness.

In their 2016 report, the Dutch Health Council advised the minister of Health, Welfare and Sport on screening and retrieval of hepatitis B and C.^11^ The Council favoured retrieval of LTFU HCV patients and indicated that this strategy is an integral part of the (after) care for diagnosed HCV patients. The advice by the Council has been endorsed by the minister of Health.

In light of all these arguments, we think that physicians have a legal and moral right to retrieve their LTFU chronic HCV patients, though they are not legally or morally bound to do so. Even though the cost-saving argument does not outweigh the patient’s autonomy in our opinion, we do not feel that retrieval threatens this autonomy. These patients have been diagnosed before and the information given during retrieval is noncommittal. The final argument in favour of a retrieval strategy is that CELINE has scientific aims. Within this scope, physicians-researchers are allowed to retrieve LTFU patients, as long as there is a clear protocol that has been approved by the appropriate regulatory bodies.

In summary, we conclude that retrieval of chronic HCV patients is possible from an ethical and/or legal perspective, when performed in a structured manner. We will now address the accompanying legal challenges.

## Legal and Ethical Issues Arising During Development of CELINE Methodology

CELINE aims to identify LTFU patients by retrospectively reviewing medical records. One of the first questions that arises is:

### 1. How far in the past is CELINE allowed to look in order to identify LTFU patients?

The Dutch Medical Treatment Contracts Act (WGBO) article 454 states that medical records should be kept for a minimum period of 15 years.^12^ However, this period can be elongated in case of chronic conditions. CELINE therefore aims to review these records as far back as possible. In other countries, the time span that caregivers should store medical records may vary. However, we would advise them to also review their records as far back as possible.

In CELINE, we chose to identify patients based on laboratory records, since the Netherlands lacks a national registry in which all diagnosed patients are registered. Using laboratory records ensures that we miss no patients, since the diagnosis is made based on blood test results. However, some countries might not be able to use laboratory records. Countries that have a national hepatitis registry, might consider identifying their patients using this database as an interface. Otherwise, the use of diagnostic coding systems, like the International Classification of Disease, might provide a good alternative.

A medical microbiologist will produce a list of all HCV tests performed. Microbiologists are allowed to share this list with other physicians, since they are regarded as a member of the treatment team according to article 457 of the WGBO,^12^ which is endorsed by the Dutch Health Council.^11^ Legislation may vary in other countries. When microbiologists are not allowed to share their list of possible LTFU patients, they could theoretically retrieve these patients themselves. However, retrieval based on only laboratory results will likely result in contacting many patients who are already cured or still in care.

The laboratory records will be reviewed in order to select patients in whom the last test result was positive, indicating that they were still infected when they left care. Hereafter, chart review is performed in order to ascertain LTFU status. This two-step selection process gives rise to the following challenge:

### 2. Who should perform selection of laboratory records and chart review in order to identify LTFU patients?

The information that has to be reviewed contains personal data, which is privileged information only divulged to members of the treatment team. Unfortunately, previous regional retrieval efforts showed that the reviewing of this data is a time-consuming process. It would be valuable if an external party could review the records and identify LTFU patients. However, this idea needs careful exploration.

The WGBO states that in order to access a patient’s medical records, the patient has to give permission.^12^ However, the WGBO has introduced an exception if obtaining permission is impossible. In the case of CELINE, hundreds of patients would have to be contacted by their treating physician to ask permission to review their medical files. This would require an extraordinary amount of time and effort. The main condition for not obtaining the patient’s permission is stated in article 458 of the WGBO: the patient’s privacy must not be disproportionately compromised. As a consequence the external party has to be trained in medical confidentiality and patient privacy. The external party must sign a confidentiality agreement prior to record review. Only data pertaining the LTFU status of the patient should be reviewed. Identifiable data should not be collected of patients who cannot be invited for re-evaluation (eg, deceased patients) or who have actively objected against the exchange of their medical records. Identifiable data of patients who object to re-evaluation should be removed. Finally, these privacy-protecting measures have to be reviewed by the institutional review board, which should give a final ruling and monitor the process.

In other countries, legislation may vary. If an external party was not allowed to review patient records without prior permission, there are several options. In any case, the laboratory records would have to be reviewed by a microbiologist first, to identify patients that might be LTFU. Subsequently there are two options. First comes the option for a member of the treatment team to review the records. The second option is to ask permission of patients to review their records. This requires pre-emptive contact with the individual patients, which presents an opportunity to offer them re-linkage to care immediately, without reviewing their medical records first. However, it is likely that many patients who are contacted are already cured or still in care.

After identification of the LTFU patients, the patient will be invited for re-evaluation.

### 3. What is the best way to invite patients?

The safest way to reach LTFU patients is by written invitation, if the sender is sure that the address is correct. In case of LTFU patients, address information might not have been updated for years. Therefore we obtain current address information from the Municipal Personal Records Database (*BasisregistratiePersonen, BRP*) when possible. Each person residing in the Netherlands for at least four months is obligated to enlist in the BRP. Organizations of public or social importance can request authorization to obtain this information, as is stated in article 3.2 of the Personal Records Database Act.^13^ Hospitals for instance request authorization in view of optimal patient care or for medical research. In hospitals that do not have access to the BRP, CELINE collaborators should make a reasonable effort to ascertain the current address for their LTFU patients. This also applies to caregivers in other countries, who cannot request address information from municipalities. We advise them to contact other healthcare providers of the patient to retrieve the patient’s address. In CELINE for example, we contact their general practitioner. If the patient is not in contact with any known healthcare provider, a test letter could be sent to the last known address, without mentioning hepatitis C. If caregivers cannot ascertain the current address for the patient, the invitation should not be sent.

## Conclusion

Retrieval of LTFU patients should only be executed when the benefits outweigh the disadvantages, as is the case in chronic hepatitis C. CELINE is the first nationwide retrieval project in the Netherlands. We have identified four major challenges of ethical and legal nature. We believe that these challenges can be translated to both HCV- and non-HCV-related retrieval projects. We have provided solutions that can be used in the Netherlands and other countries, showing that retrieval can be done when done carefully. We hope to take away any insecurities people may have about the ethical and legal nature of retrieval projects in the Netherlands and hope to inspire healthcare professionals and policy-makers in other countries to develop their own retrieval strategy that accommodates their healthcare and legal system.

## Acknowledgements

CELINE is supported with funding from Gilead Sciences.

## Ethical issues

The CMO Arnhem-Nijmegen has ruled that CELINE does not include the Medical Research Involving Human Subjects Act (WMO), and a non-WMO declaration has been provided. Each participating hospital has to provide local approval before CELINE can be initiated.

## Competing interests

FA has none to declare. MD has been a member of an advisory board of Abbvie and Gilead (fees paid to institution). JPHD declares that the Radboudumc, on behalf of JPHD, received honoraria or research grants from Novartis, Ipsen, Otsuka, Abbvie, and Gilead. JPHD served as consultant for Gilead and Abbvie, and in the last two years has been member of advisory boards of Otsuka, Norgine Gilead, BMS, Janssen, and Abbvie.

## Authors’ contributions

All authors developed concept and design of this paper. MD and FA wrote the manuscript, JD supervised and provided critical revision.

## Authors’ affiliations

^1^Department of Gastroenterology & Hepatology, Radboud University Medical Centre, Nijmegen, The Netherlands. ^2^Ethics Committee ‘CMO Arnhem-Nijmegen,’ Nijmegen, The Netherlands.
